# *Discopersicus hexagrammatus* n. sp. (Rhabditida: Tylenchidae), the second species of the genus

**DOI:** 10.21307/jofnem-2021-059

**Published:** 2021-10-26

**Authors:** Manouchehr Hosseinvand, Ali Eskandari, Joaquín Abolafia, Reza Ghaderi

**Affiliations:** 1 Department of Plant Protection, Faculty of Agriculture, University of Zanjan, 45371-38791, Zanjan, Iran; 2 Departamento de Biología Animal, Biología Vegetal y Ecología, Universidad de Jaén, Campus ‘Las Lagunillas’ s/n, Edificio B3, 23071 Jaén, Spain; 3 Department of Plant Protection, School of Agriculture, Shiraz University, 71441-65186, Shiraz, Iran

**Keywords:** 28S rDNA, Bayesian inference, Boleodorinae, *Discobasiria*, New species, Taxonomy, Tylenchidae

## Abstract

Discopersicus *hexagrammatus* n. sp., is described and illustrated from a population associated with the rhizosphere of carrot (*Daucus carota* L.) in Dezful, Khuzestan province, south-western Iran. Based on morphological and morphometric data, the new species is characterized by a 601-734 µm long body, a prominent perioral labial disc and oblique amphidial slits, 10-11 µm long stylet, vulva at 65-67% of body length, 15.5-17 µm long spicules, and an elongate conoid tail with a pointed terminus. It is the second species of the genus Discopersicus and differs from its counterpart D. iranicus, by its anterior position of vulva, shorter stylet, lower M.B. ratio, different shape of tail tip, and shorter spicules in male specimens. A molecular phylogenetic analysis using the D2-D3 expansion segments of 28S rDNA sequences, placed the new species in close relationship with five sequences of the genus *Boleodorus*.

Khuzestan province is located in southwest of Iran, and with arid and warm climate, has an important role for production of agricultural products in Iran. A large number of nematodes have been described from this province (Azimi and Pedram, 2020; Eisvand et al., 2019; Ghaderi and Karegar, 2016; Hosseinvand et al., 2019, 2020a; Panahandeh et al., 2019). Family Tylenchidae Örley, 1880 is one of the most plentiful and diverse nematode groups recovered in soil habitats, where they may represent up to 30% of the nematode abundance in any given soil sample (Qing et al., 2018). According to Karegar (2018), 115 species of this family have been reported from Iran that descriptions, morphometric data, and illustrations have been provided for 92 species of them. Afterward, 24 further species from four genera have been described (Hosseinvand, 2020).

Hosseinipour (1992) found a population of the family Tylenchidae showing a perioral labial disc and slit-like amphidial apertures, obliquely placed on lateral sides of the head. The well-known nematologist, Dr. Etienne Geraert identified it as a new genus and tentatively named it as *Discobasiria*, but the relevant paper has never been published. Hosseinvand (2014) found two species of this genus in his M.S. thesis (Karegar, 2018). Ghaemi et al. (2012) introduced the new species *Discotylenchus iranicus* (Ghaemi et al., 2012) and described it with a minute amphidial aperture, appearing as longitudinal slits in lateral view. Yaghoubi et al. (2016) restudied this species and proposed the new genus *Discopersicus*, with *D. iranicus* (Yaghoubi et al., 2016) as its type species. In this study, we describe the second species of the genus based on morphological and molecular characters.

## Materials and methods

### Nematode sampling, extracting, mounting and morphological characterization

Soil samples were collected from the rhizosphere of carrot (*Daucus carota* L.) in Dezful, Khuzestan province, southwestern Iran. Nematodes were extracted using the tray method (Whitehead and Hemming, 1965), killed and fixed by adding hot FPG (4:1:1, formaldehyde: propionic acid: glycerin), then transferred to anhydrous glycerin following the method of De Grisse (1969). To prepare nematodes for morphological observations, fixed specimens of the new species were handpicked under a Olympus SZH stereo microscope, and mounted in a small drop of pure glycerin supported with paraffin on permanent glass slides. Morphological characters were examined using a Leitz Dialux 22 light microscope. Morphometric characters and photographs were taken using a Dino-Eye digital eyepiece camera (Model AM7023, bundled with the DinoCapture 2.0 software; AnMo Electronics Corporation; New Taipei City; Taiwan) adjoined to the aforementioned microscope. Line drawings were first made using a drawing tube attached to the microscope, then redrawn and prepared for publication using CorelDRAW® software version 16.

### Scanning electron microscopy

For the scanning electron microscopy, specimens preserved in glycerin were selected for observation under SEM following the protocol by Abolafia (2015). The nematodes were hydrated in distilled water, dehydrated in a graded ethanol-acetone series, critical point dried with liquid carbon dioxide, mounted on SEM stubs, coated with gold, and observed with a Zeiss Merlin microscope (5 kv) (Zeiss, Oberkochen, Germany).

### DNA extraction, PCR, and sequencing

Nematode DNA was extracted from single live female individuals of the new species, as described by Tanha Maafi et al. (2003), and used as template for polymerase chain reaction (PCR). The D2-D3 expansion segments of 28S rDNA were amplified using the forward D2A (5′-ACAAGTACCGTGAGGGAAAGTTG-3′) and reverse D3B (5′-TCGGAAGGAACCAGCTACTA-3′) primers (Nunn, 1992). Each PCR reaction mixure with a final volume of 30 μl, contained: 15 μl Taq DNA Polymerase 2x Master Mix RED (Ampliqon, Denmark), 1 μl (10 pmol μl^−1^) of each forward and reverse primers, 2 μl of DNA template and 11 μl deionised water. Reactions were carried out in a Thermal Cycler (Hybaid, Ashford, Middlesex, UK) with an initial denaturation step of 95°C for 4 min followed by 33 denaturation cycles of 94°C for 30sec, annealing for 30 sec at 57°C, extension at 72°C for 90sec and a final extension at 72°C for 10 min. The quality of DNA targets were checked by electrophoresis of 4 μl from each of PCR products in 1% agarose gel containing ethidium bromide. The PCR products were visualized and photographed under UV light and the length of each PCR product was measured by comparison with the Low DNA Mass Ladder (Invitrogen, Carlsbad, CA, USA). The PCR products were purified and sequenced directly for both strands using the same primers with an ABI 3730XL sequencer (Bioneer Corporation, Seoul, South Korea). The newly obtained sequences were submitted to GenBank database under accession numbers MW202233 and MW202234 as indicated on the 28S phylogenetic tree.

### Phylogenetic analyses

Sequences of D2-D3 expansion segments of 28S rDNA of the new species and several representatives of the family Tylenchidae available in GenBank, were used for phylogenetic reconstruction. The newly obtained sequences were edited and aligned with another sequences available in GenBank using Muscle alignment tool implemented in the MEGA7 (Kumar et al., 2016). The ambiguously aligned parts and divergent regions were known using the online version of Gblocks 0.91b (Castresana, 2000) and were removed from the alignments using MEGA7. The best-fit model of nucleotide substitution used for the phylogenetic analysis was statistically selected using jModelTest 2.1.10 (Darriba et al., 2012). Phylogenetic tree was generated with Bayesian inference (BI) method using MrBayes 3.2.6 (Huelsenbeck and Ronquist, 2001; Ronquist et al., 2012). *Atetylenchus longilabiatus* (Hosseinvand et al., 2020a) (MN807620) and *Psilenchus hilarulus* (De Man, 1921) (EU915489) were chosen as outgroups for the tree. The analysis under general time-reversible model of sequence evolution with correction for invariable sites and a gamma-shaped distribution (GTR + I + G) model was initiated with a random starting tree and run with the Markov Chain Monte Carlo (MCMC) for 1 × 10^6^ generations. The tree was visualized and saved with FigTree 1.4.3 (Rambaut, 2014) and edited with Adobe® Acrobat® XI Pro 11.0.1.

## Results

### Systematics

#### Discopersicus hexagrammatus n. sp.

**Figure 1:**
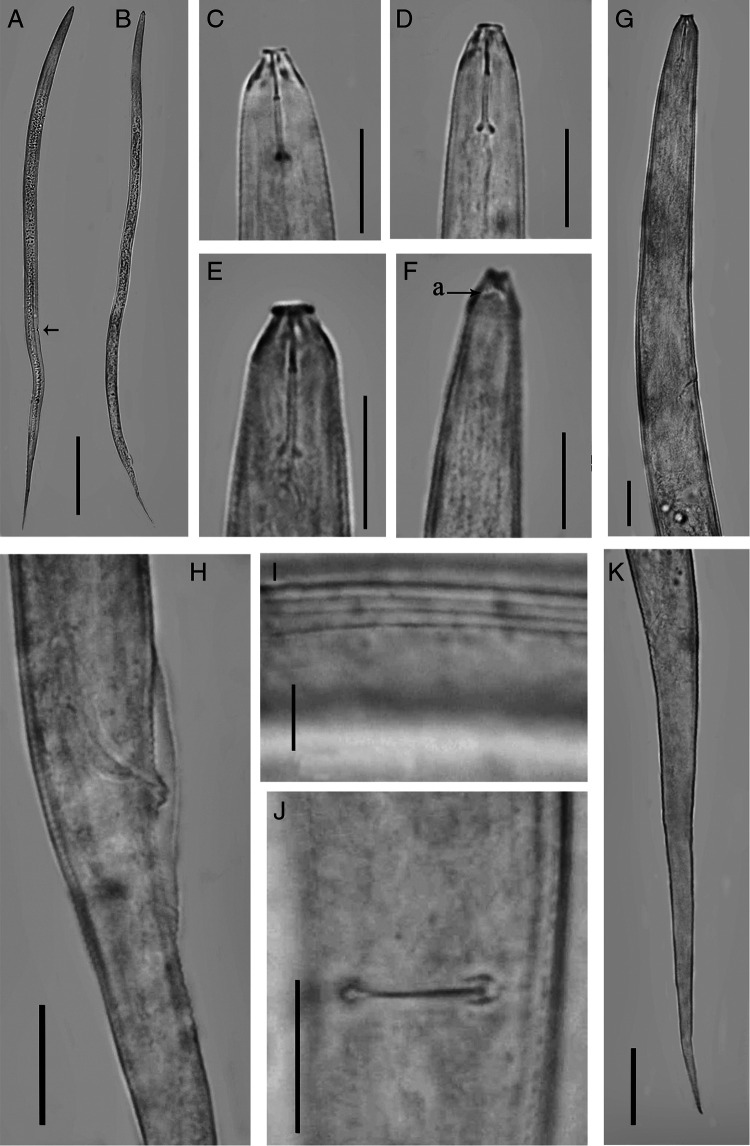
*Discopersicus hexagrammatus* n. sp. (light microscopy), female (A, C, D, F, G, I-K) and male (B, E, H). A and B: Entire body; C-E: Anterior end; F: Amphidial aperture; G: Pharyngeal region; H: Cloacal region; I: Lateral field; J: Vulval slit; K: Tail. (Scale bars: A, B = 100 µm; C-H, J, K = 10 µm; I = 5 µm).

**Figure 2:**
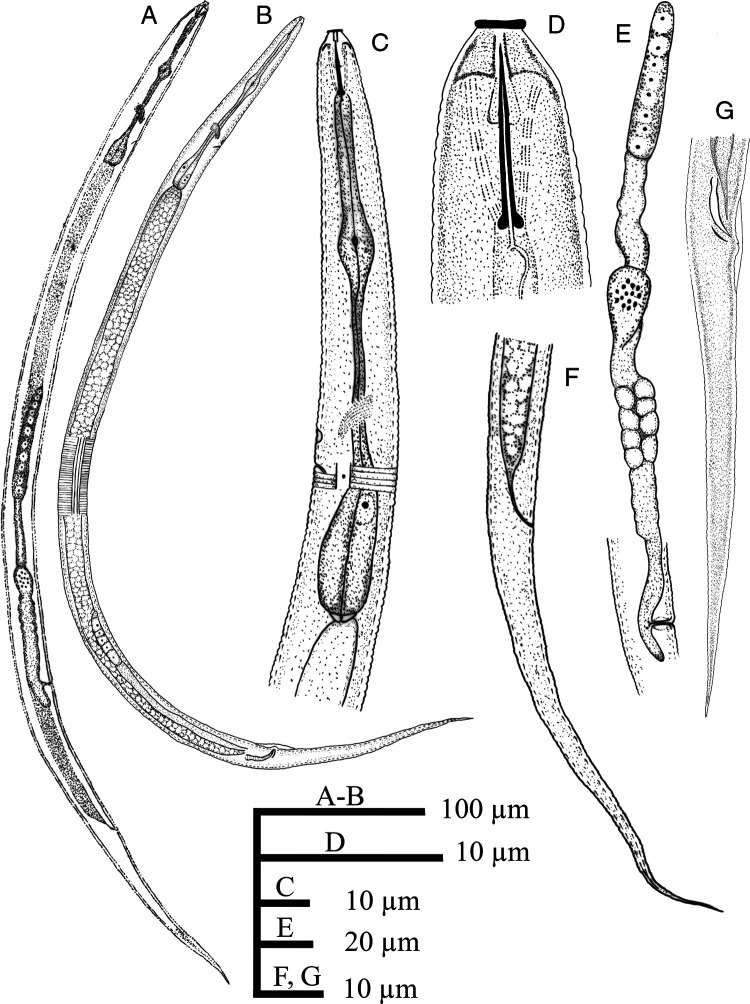
*Discopersicus hexagrammatus* n. sp. (drawing), female (A, C-F) and male (B, G). A and B: Entire body; C: Pharyngeal region; D: Anterior end; E: Reproductive system; F and G: Tail.

**Figure 3:**
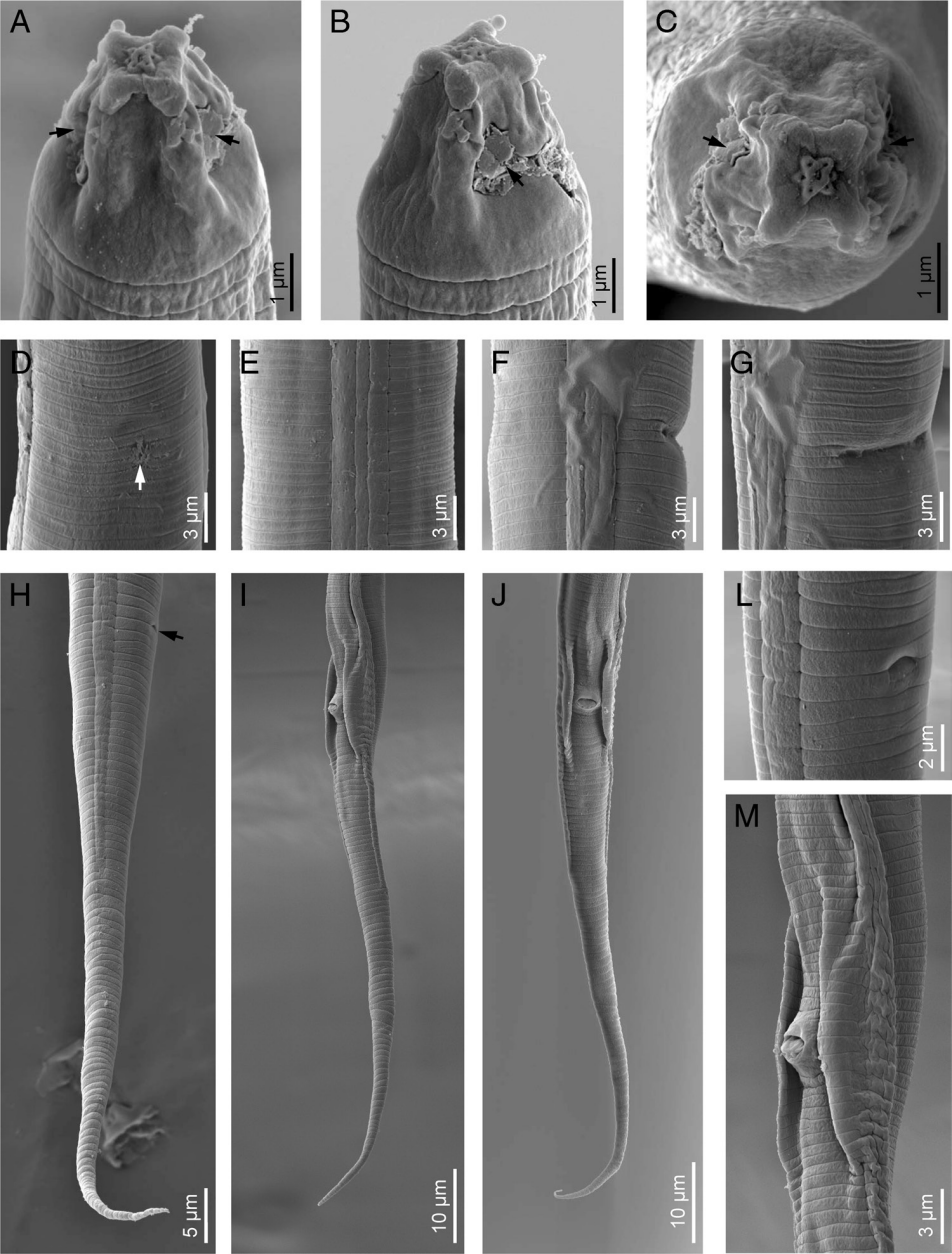
*Discopersicus hexagrammatus* n. sp. (scanning electron microscopy), female (A-H, L) and male (I, J, M). A-C: Cephalic region in lateral view (black arrows indicated amphidial apertures); D: Excretory pore in ventral view (white arrow indicated excretory pore); E: Lateral field; F and G: Vulva in ventral view; H-J: Posterior end (black arrow indicating the anus); L: Anus; M: Cloacal aperture.

(Figs. 1-3; Table 1)

**Table 1.** Morphometric data of *Discopersicus hexagrammatus* n. sp. from Iran.

	Holotype female	Paratype females		Paratype males	
*n*	–	8	CV	7	CV
L (µm)	705	688.0 ± 36.2 (612-734)	5.2	612.0 ± 8.8 (601-623)	1.4
a	37.1	35.3 ± 1.4 (34.0-37.5)	4.2	34.8 ± 2.1 (32.5-38.1)	6.1
b	6.5	6.2 ± 0.2 (5.8-6.5)	3.6	13.9 ± 0.2 (13.5-14.3)	1.8
c	11.0	10.1 ± 0.6 (9.0-11.0)	6.0	9.4 ± 0.3 (8.8-9.8)	4.0
c′	9.1	9.5 ± 0.6 (8.9-10.5)	6.7	8.4 ± 0.5 (7.6-9.0)	6.6
V	67	66.1 ± 0.7 (65-67)	1.0	–	–
V′	73	73.4 ± 1.0 (72-75)	1.4	–	–
Stylet length	10	10.3 ± 0.3 (10-11)	3.0	10.2 ± 0.3 (10-11)	3.3
m (conus/stylet %)	35	33.6 ± 1.1 (32-35)	3.4	33.1 ± 0.5 (33-34)	1.6
Anterior end to valve of median bulb	39	42.0 ± 2.2 (39-45)	5.4	40.8 ± 2.7 (38-44)	6.8
(Anterior end to pharyngeal intestinal junction) Pharynx length	108	111.0 ± 4.0 (105-117)	3.6	111.0 ± 3.5 (107-115)	3.1
M.B.	36	37.8 ± 0.9 (36-39)	2.4	36.7 ± 1.3 (35-39)	3.8
Anterior end to excretory pore	89	88.2 ± 1.8 (85-90)	2.0	86.6 ± 1.6 (84-88)	1.9
Anterior end to vulva	470	455.0 ± 22.5 (408-485)	4.9	–	–
Anterior end to anus	641	620.0 ± 33.5 (551-658)	5.4	547.0 ± 7.7 (540-557)	1.4
Vulva-anus distance	171	165.0 ± 13.2 (143-183)	8.0	–	–
Tail length/Vulva-anus distance	0.4	0.4 ± 0.1 (0.3-0.5)	10.5	–	–
Body width at midbody	19	19.5 ± 1.0 (18-21)	5.4	17.6 ± 1.1 (16-19)	6.4
Vulval body width	18	18.0 ± 0.7 (17-19)	4.1	–	–
Anal body width	7	7.1 ± 0.4 (7-8)	3.1	7.6 ± 0.2 (7-8)	2.8
Tail length	64	68.0 ± 5.2 (61-76)	7.6	64.6 ± 3.0 (61-69)	4.7
Spicules	–	–	–	16.1 ± 0.5 (16-17)	3.4
Gubernaculum	–	–	–	5.4 ± 0.2 (5-6)	4.2

**Note:** All measurements are in μm and in the form: mean ± standard deviation (range) and coefficient of variation (CV).

### Description

Female: Body straight to slightly ventrally curved. Cuticular annuli fine, 1.0 to 1.3 µm at mid-body. Lateral field with four incisures, delimiting three bands ending at middle length of tail, the inner slightly narrower than outers, not areolated, occupying 21 to 26% of the body diameter. Cephalic region smooth and flat anteriorly, with a disc-like structure in LM and a prominent offset perioral disc in SEM, 5.4 to 6.3 µm wide and 3.7 to 4.5 µm high. SEM images show the hexagram pattern of six labial papillae around oral aperture, four cephalic papillae behind the disc and amphidial apertures in the form of two oblique slits on lateral sides of cephalic region (Fig. 3A-C). Cephalic framework inconspicuous, weakly sclerotized. Stylet delicate, conus about one-third, 3.3 to 3.7 µm or 32 to 35% of the total stylet length. Dorsal pharyngeal gland opening 1.5 to 2.0 µm from stylet base. Pharynx tylenchoid with procorpus cylindroid. Pharyngeal median bulb oval with distinct valve, 39 to 45 µm from anterior end. Isthmus slender, longer than procorpus. Basal bulb pyriform to saccate-shaped, 8.0 to 10.0 µm in width and 25 to 30 µm in length. Nerve ring nearly at mid of isthmus and located at 74 to 78 µm from anterior end. Pharyngo-intestinal valve hemispherical. Excretory pore slightly sclerotized, located at anterior end of basal bulb. Hemizonid one to three annuli anterior to the excretory pore, 85 to 89 µm from anterior end. Deirids at level or slightly posterior to excretory pore, 88 to 97 µm from anterior end. Reproductive system monodelphic. Vulva with transverse slit, not protruding, without lateral flaps. Vagina 8.7 to 9.5 µm or 48 to 53% of vulva body diameter. Postvulval uterine sac short, 8.7 to 9.5 µm or 47 to 52% of vulval body diameter. Spermatheca offset, oval, 8.0 to 9.5 µm × 23 to 27 µm. Uterus perpendicular to body axis. Ovary outstretched, oocytes arranged in a single row. Rectum very short. Anus minute. Tail elongate-conoid ending to a pointed tip.

Male: General characterization similar to female except in genital system. Testis 140 to 170 µm or 24 to 31% of body length. Spicules tylenchoid-shaped. Gubernaculum simple, slightly curved. Bursa adcloacal, 20 to 25 µm long.

### Type material

Holotype, five paratype females and four paratype males were deposited in the nematode collection of the Department of Plant Protection, Faculty of Agriculture, University of Zanjan, Zanjan, Iran. Three paratype females and three paratype males were deposited in the nematode collection of the Department of Animal Biology, Plant Biology and Ecology of the University of Jaén, Spain.

### Type habitat and locality

Soil around of carrot (*Daucus carota* L.) plants in Dezful, Khuzestan Province, southwestern Iran, collected by Manouchehr Hosseinvand at February 2013 and May 2019 (GPS coordinates: 32°23′19″N, 48°28′08″E).

### Etymology

The species epithet, *hexagrammatus*, refers to the hexagram pattern of six labial papillae around the oral aperture.

### Differential diagnosis

*D. hexagrammatus* n. sp. is characterized by 601 to 734 µm body length, 10 to 11 µm stylet length, cephalic region with distinctly disc-like structure, amphidial apertures with oblique slits, tail elongate-conoid, with pointed tip and spicules 15.5 to 17.0 µm long. The new species is distinguished from the type species, *D. iranicus*, by anterior position of vulva (V = 65-67% vs 70.8-77.9%), shorter stylet (10 to 11 vs 11 to 15 µm), shorter distance from anterior end to valve of median bulb (M.B. ratio = 35-39 vs 39.1-46.0), different tail tip shape (pointed vs rounded) and shorter spicules (15.5-17.0 vs 17.5-22.0 µm).

### Molecular phylogenetic status

**Figure 4:**
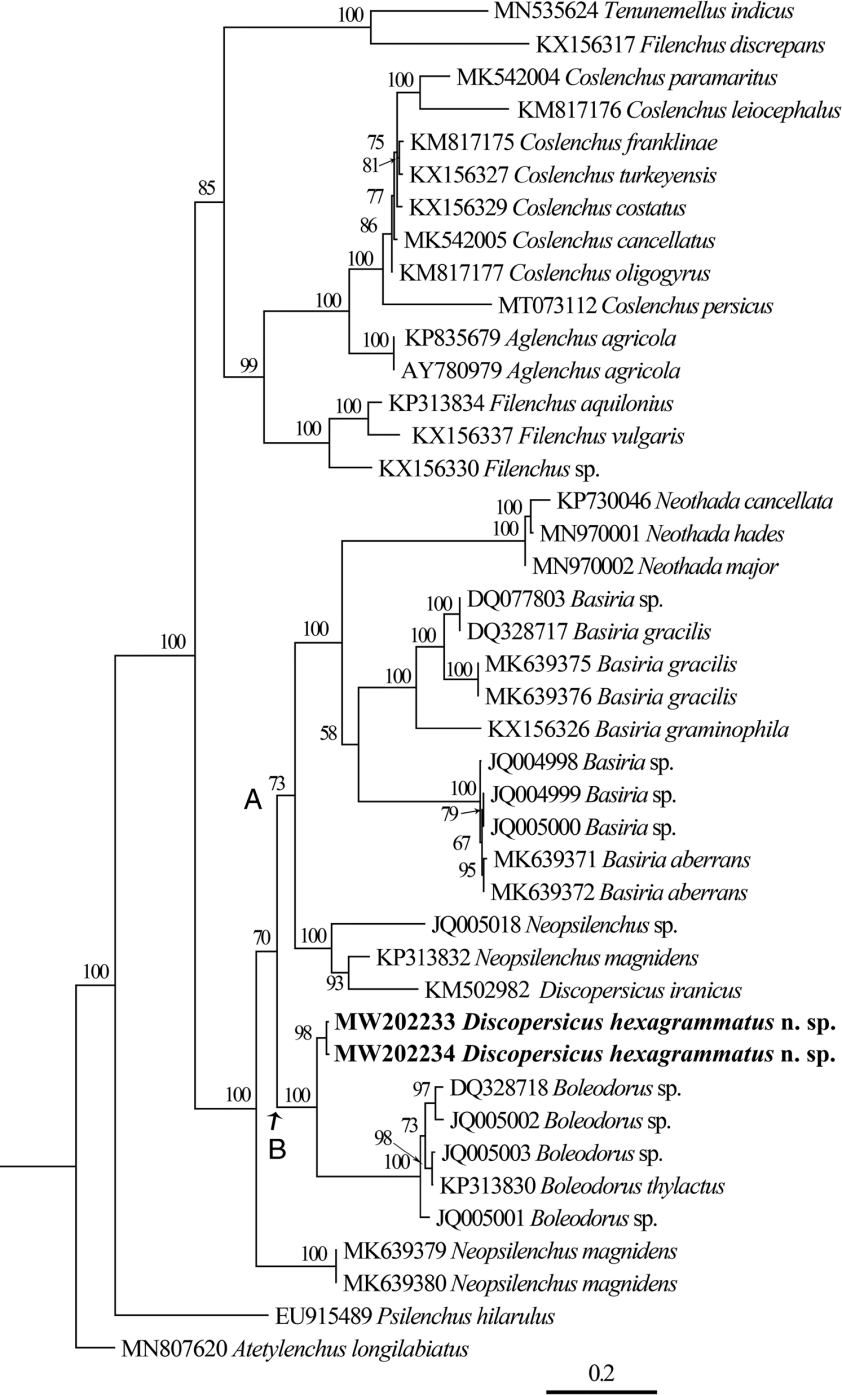
Bayesian 50% majority rule consensus tree inferred from D2-D3 expansion segments of 28S rDNA sequences of *Discopersicus hexagrammatus* n. sp. under the GTR + I + G model. Posterior probabilities more than 50% are given for appropriate clades. Newly obtained sequences in this study are in bold letters.

Amplification and sequencing of D2-D3 expansion segments of 28S rDNA from two different individuals of *Discopersicus hexagrammatus* n. sp., yielded two single fragments of 658bp long. The newly generated sequences (accession numbers: MW202233 and MW202234) showed a very low intraspecific variability with 4 different nucleotides and 0 indel (99.4% similarity). The average nucleotide compositions of the two new sequences were: (19.8, 19.8)% A; (24.5, 24.0)% T; (21.7, 21.9)% C; and (34.0, 34.3)% G, respectively. The BlastN search using these sequences showed 87% similarity (100% coverage) with Neopsilenchus magnidens (Thorne, 1949; Thorne and Malek, 1968) (KP313832, 87 different nucleotides, 578/665 identities, 19 indels), 86% similarity (99% coverage) with *Boleodorus thylactus* (Thorne, 1941) (MW716282, 92 different nucleotides, 570/662 identities, 18 indels), and 86% similarity (67% coverage) with *Discopersicus iranicus* (KM502982, 63 different nucleotides, 384/447 identities, 4 indels). The partial D2-D3 dataset for phylogenetic analysis comprised of 42 sequences, including 38 sequences of different Tylenchidae species, *Atetylenchus longilabiatus* (MN807620) and *Psilenchus hilarulus* (EU915489) as outgroups and the two newly obtained sequences. Figure 4 represents the phylogenetic tree reconstructed using this dataset. In this tree, *D. hexagrammatus* n. sp. formed a sister clade (PP = 100%) with five isolates of the genus *Boleodorus* (Thorne, 1941) (DQ328718, JQ005001–JQ005003, and KP313830). This clade has fallen into a well supported major clade formed by sequences of ten isolates of the genus *Basiria* (Siddiqi, 1959), three isolates of the genus *Neothada* (Khan, 1973), two isolates of the genus *Neopsilenchus* (Thorne and Malek, 1968) (JQ005018 and KP313832) and *D. iranicus*.

## Discussion

Our 28S rDNA tree supported the position of *Discopersicus* in the subfamily Boleodorinae (Khan, 1964) in close affinity with the genera *Basiria* and *Boleodorus*. The monophyly of the subfamily Boleodorinae was confirmed as concluded in former researches (Eisvand et al., 2019; Hosseinvand et al., 2020a, b, c; Panahandeh et al., 2019; Qing and Bert, 2018; Yaghoubi et al., 2016). The new species formed a strongly supported clade with *Boleodorus* spp., while, in Yaghoubi et al. (2016) study, *D. iranicus* was placed inside *Neopsilenchus* spp. According to the shape of spermatheca in the subfamily Boleodorinae (except for two isolates of *N. magnidens* MK639379 and MK639380 that they have different spermatheca from other members), we could observe two major clades A and B. The clade A containing species with non-offset spermatheca except *D. iranicus*, and the clade B including species with offset spermatheca. Adding the sequences of species having offset spermatheca from the genus *Basiria* can be used for testing this result.
